# N-Doped Animal Keratin Waste Porous Biochar derived from *Trapa Natans Husks*

**DOI:** 10.3390/ma13040987

**Published:** 2020-02-22

**Authors:** Wenjun Yin, Zhonghua Zhang, Tongcai Liu, Jiao Xu, Shaoze Xiao, Yao Xu

**Affiliations:** 1 State Key Laboratory of Pollution Control and Resource Reuse, College of Environmental Science and Engineering, Tongji University, Shanghai 200092, China; water_tcliu@tongji.edu.cn (T.L.); 1911421@tongji.edu.cn (J.X.); 1810827@tongji.edu.cn (S.X.); 2 UN Environment-Tongji Institute of Environment for Sustainable Development (IESD), Tongji University, Shanghai 200092, China; 3 National Engineering Research Center of Protected Agriculture, Tongji University, Shanghai 200092, China; 1833039@tongji.edu.cn

**Keywords:** animal-keratin-waste (AKWs), biochars, N/O functional groups

## Abstract

Animal-keratin-wastes (AKWs), horns (HN), hair (HR), puffed waterfowl feathers (PF), hydrolyzed waterfowl feathers (HF), hydrolyzed fish meal (HM), crab meat (CM), feathers (FR), shrimp chaff (SC), fish scales (FS), and waste leather (WL) were used as modifiers to prepare animal-keratin-wastes biochars (AKWs-BC) derived from *Trapa natans husks* (TH). AKWs-BC have a well-developed microporous structure with a pore size mainly below 3 nm. Due to the doping of AKWs, the surface chemical properties of AKWs-BC (especially N functional groups) were improved. The utilization of APWs not only realizes the resource utilization of waste, but also can be used to prepare high-performance biochars.

## 1. Introduction 

**Figure 1:**
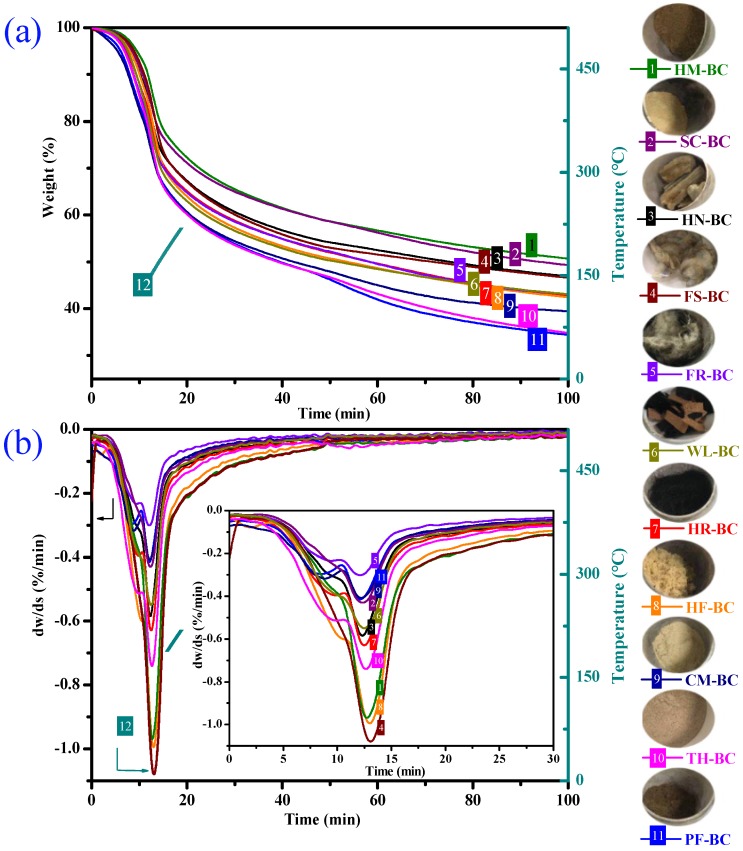
Thermogravimetric analysis (TGA) (**a**) and differential thermogravimetric analysis (DTG) (**b**) curves for the pyrolysis of animal-keratin-wastes biochars (AKWs-BC) and TH-based biochar (TH-BC).

At present, strong oxidants, nitrogen compounds, metal salts, and other chemical modifiers are mainly used for the modification of surface chemical functional groups of biochar [1,2]. However, chemical modifiers have the disadvantages of being high-cost and having secondary pollution. More than 5 million tons of animal-keratin-waste (AKWs) is produced each year in the world [3]. AKWs are single-fiber crosslinked structural proteins (S–S) with intramolecular and intermolecular disulfide bonds (S–S) [4]. AKWs represent renewable biopolymers that can be better utilized. However, there is a relative lack of information about the possibility of using AKWs as bio-modifiers to prepare biochar. During pyrolysis, the formation of novel radicals is produced by AKWs decomposition, which can react with precursors to produce certain new ester salts and promote the formation of new functional groups. Therefore, we used ten common AKWs (Table 1 and Figure 1), including horns (HN), hair (HR), puffed waterfowl feathers (PF), hydrolyzed waterfowl feathers (HF), hydrolyzed fish meal (HM), crab meat (CM), feathers (FR), shrimp chaff (SC), fish scales (FS), and waste leather (WL), as modifiers to obtain environmentally sustainable biochar with a high surface chemistry.

## 2. Experimental Materials and Procedures

Preparation method: All chemicals used were of analytical grade. *Trapa natans* (TH), containing a large amount of lignin and cellulose, is high-yield waste. TH-based biochar has well developed pore structure and specific surface area. TH (Taihu in Jiangsu, China) and ten groups of AKWs (from one farmers’ market in Shanghai, China) were crushed into particle between 0.35 and 1.0 mm. TH was mixed with 10 groups of AKWs, respectively, and the mixed mass ratio was 99:1 (g TH/g AKWs). Each mixture was impregnated with 85% phosphoric acid (g phosphoric acid/g TH = 2.2:1) for 10 hours at room temperature. To improve the degree of impregnation, series of pretreatments were used to treat the AKWs: the samples were first soaked in PPA (phosphoric acid, 85 wt.%) for 30 min, then irradiated with ultrasound for 20 min, and finally heated at 100 °C and 1.5 MPa for 30 min in a vertical automatic electrothermal pressure steam sterilizer. Each of the impregnation slurries was heated to 500 °C (heating rate = 100 °C/min) in a tube furnace and maintained at this temperature for 1 hour under nitrogen protection. Each carbon solid was washed several times with clean water until the pH of the filtrate was near neutral (about 7). Each carbon solid was dried, ground, and sieved (160–200 mesh). Finally, 10 sets of biochar were obtained: HN-BC, HR-BC, PF-BC, HF-BC, HM-BC, CM-BC, FR-BC, SC-BC, FS-BC, and WL-BC, which were collectively called AKWs-BC. Pure TH-based biochar (TH-BC) was prepared by the same method in Table 1 and Figure 1.

Characterization methods: surface area and pore size distribution were determined by N_2_ adsorption-desorption at 77 K with a surface area analyzer (Quanta Chrome Corporation, Mahwah, NJ, USA). Sur-face area (S_BET_) was measured by the BET (Brunauer–Emmet–Teller equation) method. Pore size distribution was determined by the density functional theory (DFT) method. Micropore volume (V_mic_) and micropore surface area (S_mic_) were calculated using the t-plot method. The total pore volume (V_tot_) was deduced from the manufacturer’s software by the BJH theory. The contents of C, H, O, N, and S of the biochar were measured by a Vario EI III Element Analyzer (Mahwah, NJ, USA). Boehm titration method was used to quantify the acidic and basic functional groups of the biochars. XPS (X-ray photoelectron spectroscopy) analyzer (Nico-let-460, Thermo Fisher, Mahwah, NJ, USA) was conducted to determine the binding energy between electrons and characterize the elements on the surface of biochars.

## 3. Experimental Results and Discussion

Thermogravimetric analysis (TGA) and differential thermogravimetric analysis (DTG) curves for the pyrolysis of AKWs-BC and TH-BC were obtained by TGA-50 analyzer (Figure 1). As shown in the Figure 1, the pyrolysis process of each sample is roughly divided into three decomposition stages. In stage 1 (0–140 °C), AKWs-BC’s weightlessness was greater than that of TH-BC, which can be explained by the first metamorphic decomposition of keratin [5]. Stage 1 also involved elimination of water vapor and other volatile substances. In stage 2 (140–500 °C), the new radicals produced by keratin decomposition formed different salt and esters with the thermal hydrolysate of TH and the heat activator, thereby promoting the formation of functional groups on the surface of the biochar. In stage 3 (500 °C), the weightlessness of each sample was not significant, indicating formation of the basic structure of biochar.

**Figure 2:**
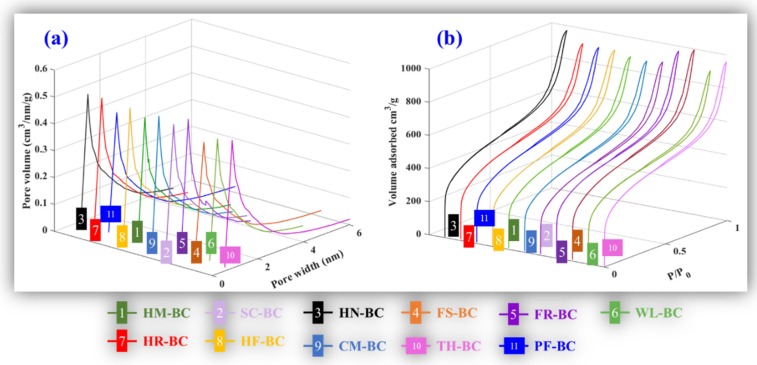
The pore size distributions (**a**) and N_2_ adsorption/desorption isotherms (**b**) of the AKWs-BC and TH-BC.

The pore size distributions (Figure 2a); N_2_ adsorption/desorption isotherms (Figure 2b); and textural properties of S_BET_, S_mic_, V_mic_, and V_tot_ (Table 1) of AKWs-BC and TH-BC were obtained by automatic specific surface area and pore size analyzer (GEMINT VII 2390, Mahwah, NJ, USA). As shown in the Figure 2a, both APWs-BC and TH-BC have a narrow pore size distribution (2–3 nm). As shown in the Figure 2b, the isotherms of AKWs-BC and TH-BC were mixture of types I and IV (IUPAC), with small hysteresis loop indicating the presence of well-developed mixed micro-mesopores structure. The variation coefficients in the S_BET_, S_mic_, V_mic_, and V_tot_ groups were analyzed by SPSS software (Table 1). The low dispersion state of the data within each group indicates that the pore structure characteristics of AKWs-BC and TH-BC are similar.

Boehm’s titration results and element composition of the AKWs-BC and TH-BC are shown in Table 1. The percentage content of O and N elements in AKWs-BC is increased compared to TH-BC, representing the increase in the content of acidic/basic functional groups on the surface of AKWs-BC. The number of acidic and basic functional groups on the carbon’ surface of AKWs-BC were about 2.2 and 1.2 times that of TH-BC, respectively, which suggested that AKWs facilitated the creation of acidity and basicity on the surface of AKWs-BC because AKWs contain a large amount of N and O. 

**Figure 3:**
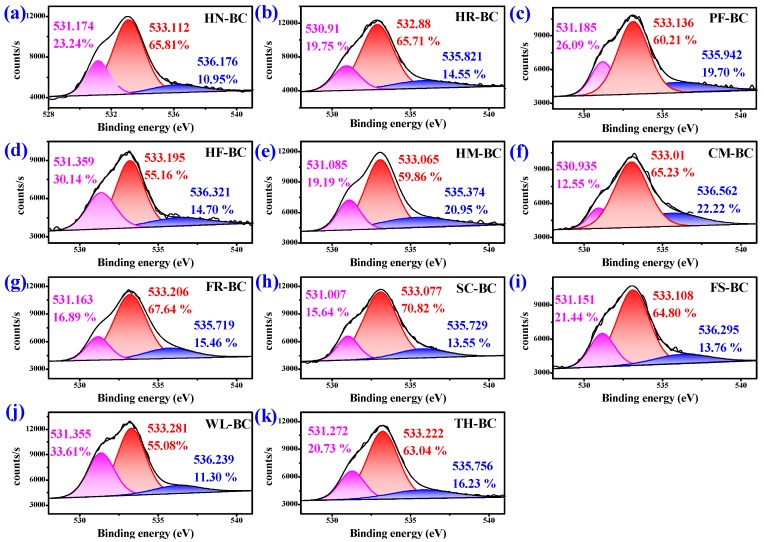
XPS (O 1s) spectra of AKWs-BC and TH-BC: HN-BC (**a**); HR-BC (**b**); PF-BC (**c**); HF-BC (**d**); HM-BC (**e**); CM-BC (**f**); FR-BC (**g**); SC-BC (**h**); FS-BC (**i**); WL-BC (**j**); TH-BC (**k**).

**Figure 4:**
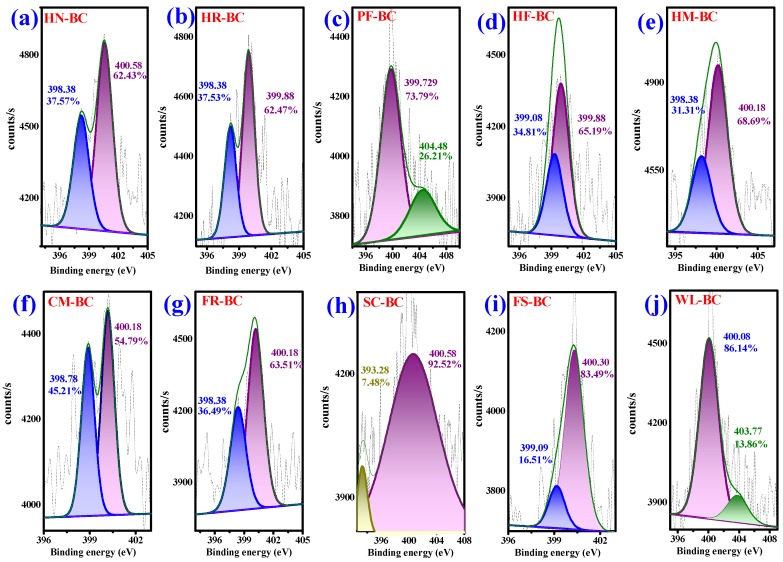
XPS spectra (N 1s) of AKWs-BC: HN-BC (**a**); HR-BC (**b**); PF-BC (**c**); HF-BC (**d**); HM-BC (**e**); CM-BC (**f**); FR-BC (**g**); SC-BC (**h**); FS-BC (**i**); WL-BC (**j**).

XPS was used to identify surface functional groups of AKWs-BC and TH-BC. The peak fitting of O 1s and N 1s was performed by XPS-PEAK software. The oxygen functional groups on the surface of AKWs-BC exhibit three peaks in Figure 3: (O-I) C=O groups (carbonyl and quinone) at 531.1 ± 0.5 eV, (O-II) C-OH/C-O-C (hydroxyl ether ester and anhydride) at 533.1 ± 0.5 Ev, and (O-III) –COOH at 535.8 ± 0.5 eV. The nitrogen functional groups on the surface of AKWs-BC presented two peaks in Figure 4: (N 6, Pyridine nitrogen and amino group) –CONH– / N–H at 398.7 ± 0.5 eV or (N 5, pyrrole-like nitrogen) –CONH–/–NH_2_ at 399.8 ± 0.5 eV, while –NO_2_ represents nitrogen in nitro group (403.6–405.1 eV) [6,7]. The number of oxygen-containing and nitrogen-containing functional groups of AKWs-BC is higher than that of TH-BC, which is consistent with the results of Boehm’s titration results and element composition. Overall, there is strong evidence that APWs promote the formation of functional groups on the surface of the AKWs-BC.

## 4. Conclusions

The results show that it is feasible to use AKWs as a biochar modifier. AKWs-BC exhibits a developed microporous structure and high chemical functional groups. The use of APWs can not only realize the resource utilization of waste, it can also provide new ideas for biochar preparation modification.

## Figures and Tables

**Table 1:** Textural parameters, amount of acidic and basic functional groups, and elemental compositions of the AKWs-BC and TH-BC.

AKWs	Biochars	S_BET_ (m^2^/g)	S_mic_ (m^2^/g)	V_tot_ (cm^3^/g)	V_mic_ (cm^3^/g)	Acidic (mmol/g)	Basic (mmol/g)	C (%)	N (%)	O (%)	S+H (%)
HN	HN-BC	1503	491	1.51	0.20	3.283	1.821	73.47	1.4	22.22	2.91
HR	HR-BC	1422	443	1.41	0.18	3.358	1.832	72.81	1.42	23.38	2.39
PF	PF-BC	1403	424	1.40	0.17	3.382	1.803	73.42	1.33	23.42	1.83
HF	HF-BC	1331	354	1.40	0.15	3.183	1.812	75.03	1.33	21.49	2.15
HM	HM-BC	1342	380	1.37	0.16	3.154	1.805	75.53	1.31	21.16	2.00
CM	CM-BC	1301	358	1.36	0.15	3.503	1.672	71.81	1.11	24.85	2.23
FR	FR-BC	1317	365	1.38	0.15	3.32	1.677	74.15	1.12	23.13	1.6
SC	SC-BC	1376	389	1.51	0.20	3.098	1.712	75.32	1.16	21.43	2.09
FS	FS-BC	1503	438	1.55	0.17	3.294	1.752	73.93	1.28	22.69	2.1
WL	WL-BC	1434	445	1.38	0.20	3.412	1.811	72.71	1.39	23.99	1.91
-	TH-BC	1492	491	1.49	0.20	1.426	1.423	72.38	0.42	16.35	10.85
**variation coefficients**	*c_v_*	0.051	0.115	0.0444	0.117	-	-	-	-	-	
BET surface area (S_BET_), micropore surface area (S_mic_), micropore volume (V_mic_), total pore volume (V_tot_).											
